# Tenascin C promotes valvular remodeling in two large animal models of ischemic mitral regurgitation

**DOI:** 10.1007/s00395-020-00837-5

**Published:** 2020-12-01

**Authors:** Ouafa Hamza, Attila Kiss, Anne-Margarethe Kramer, Sandra Trojanek, Dietmar Abraham, Eylem Acar, Felix Nagel, Verena Eva Tretter, Melitta Kitzwögerer, Bruno K. Podesser

**Affiliations:** 1 Ludwig Boltzmann Institute for Cardiovascular Research at the Center for Biomedical Research, Medical University of Vienna, Waehringer Guertel 18-20, 1090 Vienna, Austria; 2 Center for Anatomy and Cell Biology, Medical University of Vienna, Vienna, Austria; 3 Department of Cardiac Surgery, Karl Landsteiner University, St. Pölten, Austria; 4 Department of Anesthesia, General Intensive Care and Pain Therapy, Medical University of Vienna, Vienna, Austria; 5 Department of Pathology, Karl Landsteiner University, St. Pölten, Austria

**Keywords:** Ischemic mitral regurgitation, Myocardial infarction, Leaflet remodeling, Tenascin C, Endothelial-to-mesenchymal transition

## Abstract

Ischemic mitral regurgitation (MR) is a frequent complication of myocardial infarction (MI) characterized by adverse remodeling both at the myocardial and valvular levels. Persistent activation of valvular endothelial cells leads to leaflet fibrosis through endothelial-to-mesenchymal transition (EMT). Tenascin C (TNC), an extracellular matrix glycoprotein involved in cardiovascular remodeling and fibrosis, was also identified in inducing epithelial-to-mesenchymal transition. In this study, we hypothesized that TNC also plays a role in the valvular remodeling observed in ischemic MR by contributing to valvular excess EMT. Moderate ischemic MR was induced by creating a posterior papillary muscle infarct (7 pigs and 7 sheep). Additional animals (7 pigs and 4 sheep) served as controls. Pigs and sheep were sacrificed after 6 weeks and 6 months, respectively. TNC expression was upregulated in the pig and sheep experiments at 6 weeks and 6 months, respectively, and correlated well with leaflet thickness (*R* = 0.68; *p* < 0.001 at 6 weeks, *R* = 0.84; *p* < 0.001 at 6 months). To confirm the translational potential of our findings, we obtained mitral valves from patients with ischemic cardiomyopathy presenting MR (*n* = 5). Indeed, TNC was also expressed in the mitral leaflets of these. Furthermore, TNC induced EMT in isolated porcine mitral valve endothelial cells (MVEC). Interestingly, Toll-like receptor 4 (TLR4) inhibition prevented TNC-mediated EMT in MVEC. We identified here for the first time a new contributor to valvular remodeling in ischemic MR, namely TNC, which induced EMT through TLR4. Our findings might set the path for novel therapeutic targets for preventing or limiting ischemic MR.

**Electronic supplementary material:**

The online version of this article (10.1007/s00395-020-00837-5) contains supplementary material, which is available to authorized users.

## Introduction

Secondary Mitral regurgitation (MR) is a frequent complication of myocardial infarction (MI) that worsens patients' prognosis [19]. Intricate mechanisms can be identified at the origin of the ischemic MR. As the left ventricle (LV) undergoes remodeling, the papillary muscle displacement causes mitral leaflet tethering resulting in systolic restriction known as type III from Carpentier classification. Also, the progressive annulus dilatation jeopardizes the MV coaptation representing an additional mechanism classified as Type I according to Carpentier.

The mitral valve (MV) can adapt to mechanical stress. As the LV undergoes remodeling with cavity enlargement, the MV leaflets area increases to limit and reduce MR [23]. This area increase is not only achieved by simple passive stretch, but also is the result of endothelial cell activation through endothelial-to-mesenchymal transition (EMT) [6]. Furthermore, it has been shown that MV generates different responses to tethering forces in the presence or absence of an ischemic myocardial environment [7]. Indeed, EMT, which is an early developmental process [5, 15], is reactivated during ischemia in a more-sustained manner resulting in stiff leaflets [7].

Tenascin C (TNC), an extracellular matrix (ECM) glycoprotein, is transiently expressed at the embryonic development stage, but is usually not expressed in healthy adult hearts. It is upregulated post-MI and associated with cardiac remodeling [11, 21]. TNC has been shown to induce epithelial-to-mesenchymal transition enhancing the migration potential of malignant cells [14]. In addition, TNC is also known to react to mechanical stress [25]. It has also been incriminated in aortic stenosis pathophysiology and its evolution [24].

TGFβ, an accepted EMT inducer, has been shown to contribute to excess valvular EMT and remodeling in ischemic MR [7]. However, no previous data are available regarding TNC role in ischemic MR at the valvular level nor its possible contribution to adult valvular EMT.

In this study, we hypothesized that TNC induces endothelial-to-mesenchymal transition and subsequently promotes mitral valve remodeling. For these purposes, we designed a short- and long-term ischemic MR model, in pig and sheep, respectively, to study the valvular remodeling occurring at an early and late stage of ischemic MR and its possible correlation to TNC expression. We also used in-vitro experiments to investigate whether TNC could induce EMT in isolated mitral valvular endothelial cells (MVEC).

## Materials and methods

### Large animal models of ischemic mitral regurgitation

Data from 14 young female Landrace domestic pigs (> 45 kg) and 11 adult female sheep (> 70 kg) were collected. Ischemic MR was achieved by ethanol injection in the obtuse marginal branches (OM) of the circumflex artery responsible for the posteromedial papillary muscle (PMPM) vascularization identified by contrast echocardiography as previously described [8]. The animal experiments were approved by the local ethics committee (BMWFW-66.009/0112-WF/V/3b/2017) and conform to the NIH guidelines for animal care.

First, an ischemic MR model was established in 7 pigs, which were sacrificed 6 weeks later, while healthy pigs (*n* = 7) were used as controls. The pigs' experiments previously reported [8] now have extensive additional analyses. We then wanted to observe the characteristics of the valvular remodeling over a longer period of ischemic MR. Unfortunately, the pig presents the limitation of its rapid growth rate. This is why, we enrolled sheep in long-term follow-up experiments (6 months) and assigned to an ischemic MR group (*n* = 7) and a control group (*n* = 4). Anesthetic regiments and periprocedural therapy are described in Supplementary Table 1.

### Echocardiography

LV end-diastolic, end-systolic diameters (EDD and ESD) were obtained from short- and long-axis M-Mode by averaging three cycles in each view. Left ventricle ejection fraction (LVEF), and end-diastolic and end-systolic volumes were measured in two-dimensional (2D) mode using the Simpson´s method. Color Doppler was used to evaluate MR severity by measuring the jet surface, the indexed jet area to the left atrium (IJA), and the vena contracta as previously described [26]. Tenting area was measured as the surface below the mitral anulus plane in mid-systole, while the tenting height represented the distance between the leaflet coaptation and the mitral anulus plane at mid-systole.

Leaflet length was measured by two-dimensional echo from leaflet insertion to tip in the parasternal long-axis view while in diastasis before end diastole.

### Histology

**Fig. 1:**
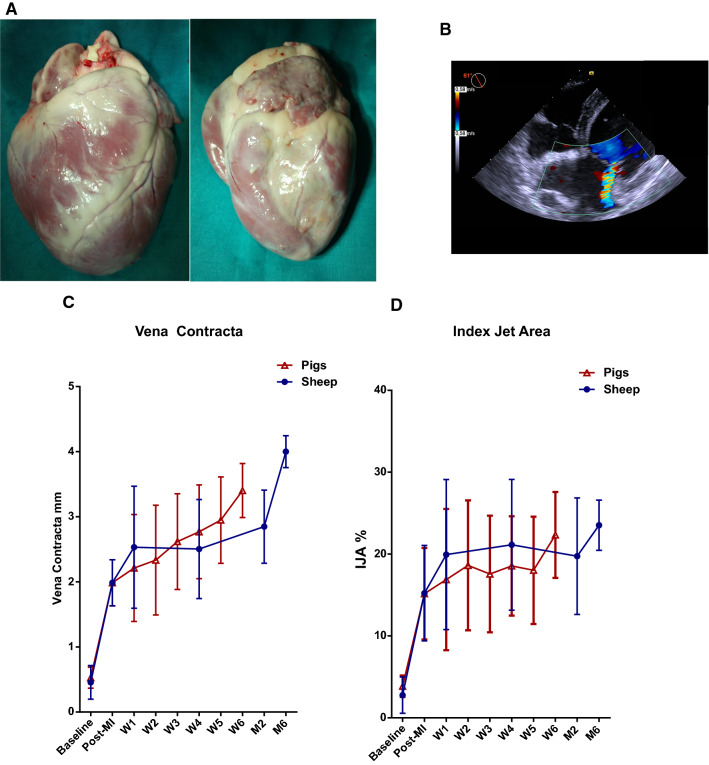
Ischemic MR animal model. Harvested heart arrested in diastole after potassium overdose anterior (left) and posterior (right) views (**a**). Representative echocardiogram with color Doppler 6 months post-MI in a sheep (**b**). Mitral regurgitation severity temporal evolution was evaluated using vena contracta (**c**) and index jet area (**d**)

Pigs and sheep were euthanized after 6 weeks or 6 months, respectively, and the hearts harvested (Fig. 1a). Anterior and posterior mitral leaflets were dissected and collected for histology. Tissue samples were fixed in formalin and paraffin-embedded. Leaflet thickness was measured in midportion of the leaflets. TNC, CD31, α-smooth muscle actin (α-SMA), and Toll-like receptor 4 (TLR4) immunohistochemical stainings were performed by Streptavidin Biotin method (Supplementary Table 2).

Besides, we obtained mitral valve specimens from patients suffering from ischemic cardiomyopathy and undergoing surgical valve intervention for severe functional MR (n = 5) and were stained for TNC. Informed consent was obtained from all patients (Ethical approval under GS1-EK-4/288–2014, ethical commission of Lower Austria, St. Pölten, Austria).

### Cell culture

Mitral valve endothelial cells (MVEC) were isolated from healthy porcine hearts as previously described [3]. Briefly, excised porcine mitral valves were collected in DMEM and were transported on ice to the cell culture laboratory where the isolation under aseptic conditions was conducted. The valves were incubated with the Collagenase II solution for 10 min at 37 °C. The isolated cells were plated on rat tail Collagen1 (5 µg/cm^2^) precoated dishes and supplemented with EC medium (Dulbecco's Modified Eagle Medium, 10% fetal bovine serum, 1% Penicillin–Streptomycin, and Heparin 50 UI/ml). To sort MVEC, the cells were labeled with a CD31 rabbit polyclonal anti-pig antibody (abcam ab28364) and were separated using Anti Ig G rabbit magnetic micro-beads. The MVEC were plated at a density of 10,000 cells/cm^2^. After 24 h, the medium was replaced by fresh endothelial cell (EC) medium supplemented with recombinant human Tenascin C (1 or 5 µg/ml) and were incubated for either 24 or 48 h before being harvested for RT-qPCR analysis. In addition, transforming growth factor-β1 (TGFβ1) was used as positive control, since it is known to induce EMT in MVEC [6]. To that end, MVEC were cultured in the presence of human TGFβ1 (1 ng/ml) and were harvested after 96 h for RT-qPCR analysis as previously described [1].

### TLR4 inhibition assay

MVEC were plated at a density of 10,000 cells/cm^2^ and were allowed to attach for 24 h. Cells were pretreated with a specific TLR4 inhibitor (TAK242 50 nM) and the medium was replaced 30 min later by fresh EC medium containing either TGFβ1 (1 ng/ml) or hTNC (5 µg/ml). The cells were harvested for RT-qPCR analysis after 96 h of TGFβ1 or 48 h of hTNC stimulation.

### Quantitative polymerase chain reaction (RT-qPCR)

Total cellular RNA was isolated using RNeasy Mini kit (Qiagen, Hilden, Germany). Reverse transcriptase reactions were performed using QuantiTect reverse transcription kit (Qiagen). Quantitative polymerase chain reaction was performed using qPCR and was performed using QuantiTect SYBR Green PCR kit (Qiagen, Hilden, Germany). Amplification was performed in a Rotor-Gene Q (Qiagen, Hilden, Germany). A standard curve for each gene was generated to determine amplification efficiency. The primers used for the RT-qPCR are listed in Supplementary Table 3. HMBS and TBP were used as housekeeping genes expression reference. Each amplification reaction was performed in duplicates. Relative gene expression was calculated by 2^−ΔΔCt^ method.

### Statistical analysis

All results are expressed as means ± standard deviation (SD). Sample variances were analyzed by Fisher’s tests to determine equal or nonequal variances. A Fisher *p* value > 0.05 was considered equal variance. The difference between two variables at different time points was performed using a repeated-measured two-way ANOVA with a post hoc Tukey´s test. Comparison between groups was performed using an unpaired *t* test. *p* values < 0.05 were considered significant. Statistical analysis was done using Prism^TM^6 software (GraphPad Inc., San Diego, CA, USA).

## Results

### Animal characteristics

Body weight at euthanasia was in the pigs: 90 ± 15 kg in the ischemic MR group vs 85 ± 7 kg in control animals; *p* = 0.27 and in the sheep: 96 ± 9 kg in the ischemic MR group vs 86 ± 14 kg in control animals; *p* = 0.11.

Both models showed a localized PMPM infarct resulting in a moderate ischemic MR with reduced LVEF (Fig. 1). PMPM infarct induced valvular tethering as shown by the increase in tenting area (Supplementary Table 4).

### Mitral valve remodeling

**Fig. 2:**
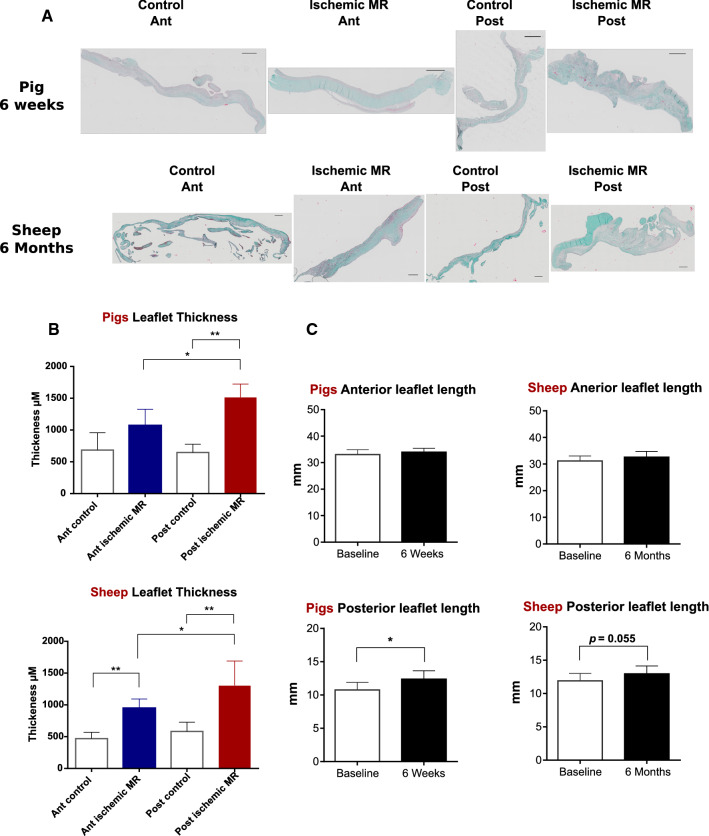
Valvular remodeling in pig and sheep models. Masson’s trichrome staining of anterior (Ant) and posterior (Post) mitral leaflet in 6 weeks (pigs) and 6 months (sheep) groups scale bar 500 µm (**a**). Anterior and posterior leaflet thickness 6 weeks and 6 months after ischemic MR in comparison to control animals; *p < 0.01, **p < 0.001 (**b**). Echocardiographic measurements of anterior and posterior mitral leaflet length in ischemic MR animals after 6 weeks (pigs) and 6 months (sheep) compared to control animals. *p < 0.05 (**c**)

Posterior leaflet length was significantly increased after 6 weeks of ischemic MR compared to controls, but there was no significant difference between ischemic MR and control animals after 6 months in the sheep experiments. Anterior leaflet length did not increase neither after 6 weeks nor 6 months (Fig. 2c).

A significantly increased posterior leaflet thickness was observed after 6 weeks and 6 months compared to control animals. Furthermore, the posterior leaflet was significantly thicker than the anterior leaflet among the ischemic MR pigs. In addition, the sheep presented a significant increase in the anterior leaflet thickness after 6 months of ischemic MR compared to control animals (Fig. 2a, b).

### Leaflet TNC expression in the ischemic MR models (Fig. 3)

**Fig. 3:**
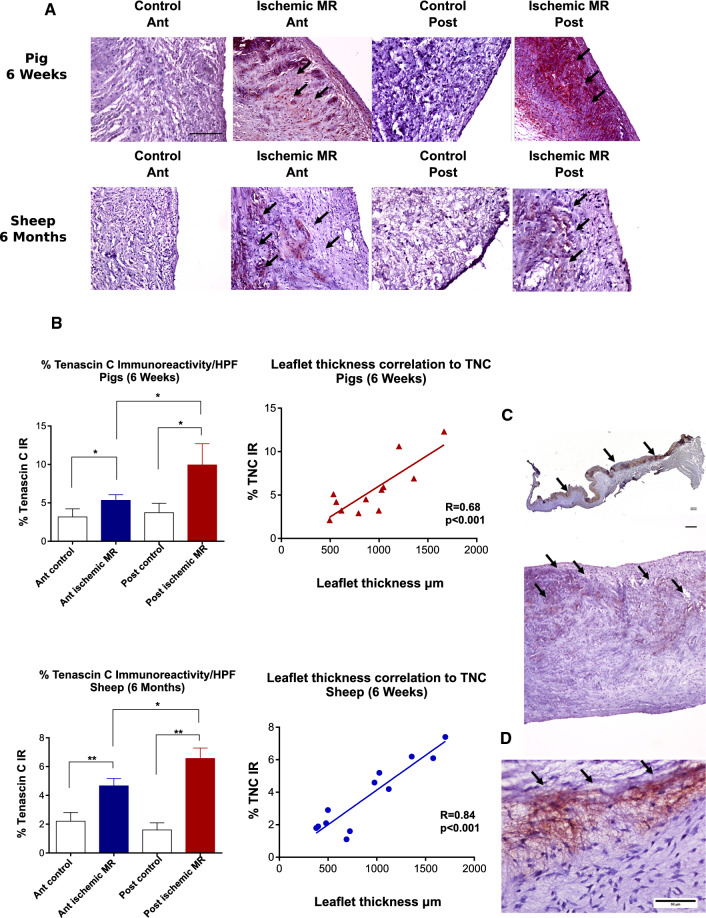
Leaflet TNC expression in the ischemic MR animal models and patients’ samples. Tenascin C (TNC) staining (black arrows) of anterior (Ant) and posterior (Post) mitral leaflet. Scale bar 100 µm (a) Quantitative analyses of TNC staining and its correlation to leaflet thickness; *p < 0.01, **p < 0.001 (b). TNC staining (black arrows) was mostly present in the atrial side of the posterior mitral leaflet in the pigs (upper panel) and in the sheep (lower panel); (c) representative TNC staining of mitral leaflet from human patient with ischemic MR (d)

TNC expression was not detected in mitral leaflets from healthy animals, while a prominent TNC expression was observed in the ischemic MR groups. Six weeks post-MI, TNC expression was more significant in the posterior leaflet. In the sheep experiments, TNC was also markedly expressed in the posterior leaflet compared to the anterior leaflet in the ischemic MR animals (Fig. 3a). In addition, TNC expression at valvular level correlated well with the leaflet thickness in both pigs and sheep groups (Fig. 3b). We also noted that TNC expression was more pronounced on the atrial side of the leaflets (Fig. 3c).

### TNC expression in mitral leaflets from patients with ischemic MR (Fig. 3d)

To prove the translational potential of our findings from the animal models, we performed TNC staining in mitral leaflets obtained from patients with ischemic MR (*n* = 5). The patients (4 males and 1 female with a mean age of 70.8 ± 9.3 years) had a severe ischemic MR with a mean LVEF of 48 ± 4% and LV EDD 59 ± 12 mm. Most importance, TNC expression was also observed in all 5 patients’ samples similar to findings in the large animal models.

### EMT and TLR4 expression

**Fig. 4:**
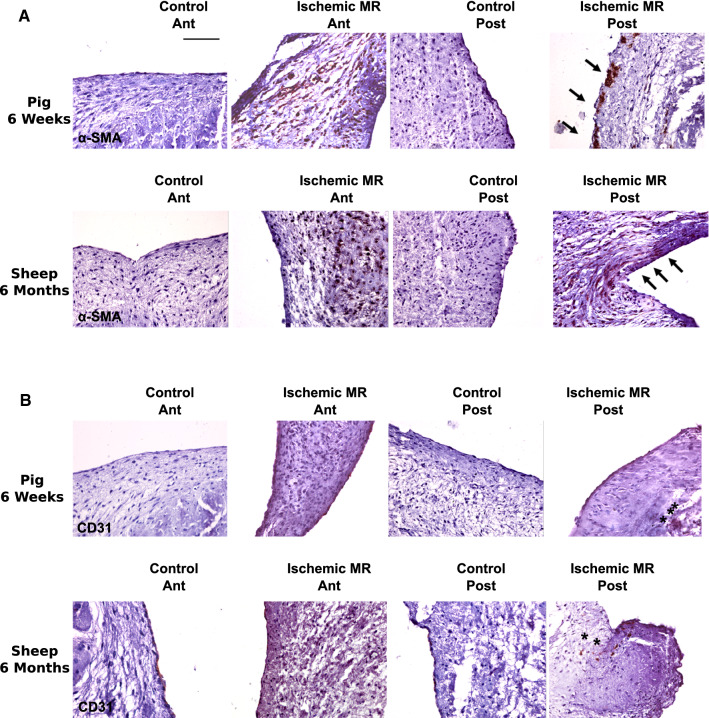
Indirect signs of EMT in ischemic animal MR models. α-SMA (**a**) and CD31 (**b**) staining of anterior (Ant) and posterior (Post) mitral leaflet. Scale bar 50 µm

Non-activated endothelial cells in their basal state express CD31 but not α-SMA, whereas endothelial cells undergoing EMT express α-SMA, while they lose CD31 expression. In both our models (6 weeks and 6 months follow-up), we observed the presence of cells expressing α-SMA in the endothelium (black arrows) as well as an interstitial invasion of α-SMA positive cells (Fig. 4a). Interestingly, we observed in the posterior leaflet 6 months post-MI the presence of CD31 cells not only along the endothelial layer but also in the depth of the leaflet interstitium (black stars, Fig. 4b). These findings suggest a valvular EMT process.

**Fig. 5:**
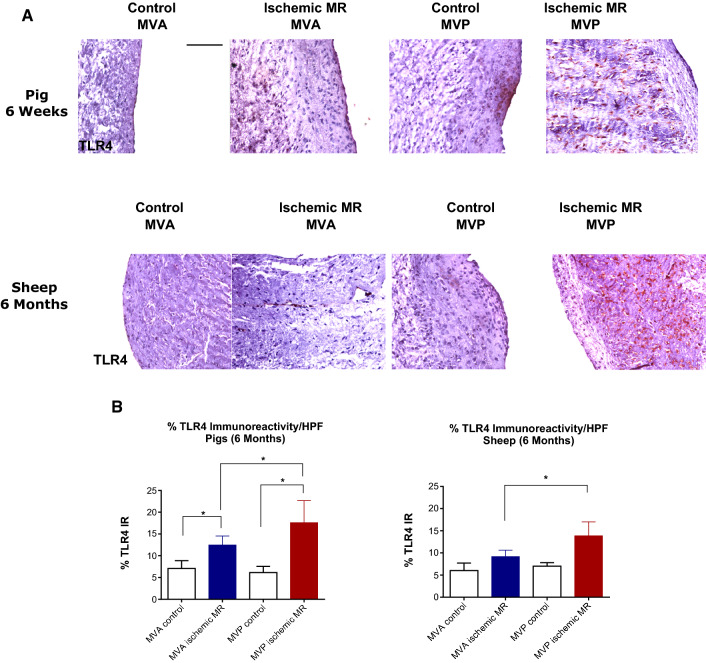
TLR4 valvular expression in ischemic MR animal models. TLR4 staining of anterior (Ant) and posterior (Post) mitral leaflet. **a** Quantitative analyses of TLR4 staining; *p < 0.01 (**b**)

We also investigated the extent of TLR4 expression as inflammatory surrogate. In control leaflets, TLR4 was mostly expressed by the cells localized in the endothelial layer, whereas in ischemic MR leaflets, cells expressing TLR4 were localized in the leaflet interstitium (Fig. 5a). Although cells expressing TLR4 were present in the interstitium of both leaflets after 6 weeks and 6 months post-MI, their number was significantly higher in the posterior leaflet (Fig. 5b).

### TNC induces EMT in MVEC (Fig. 6a)

**Fig. 6:**
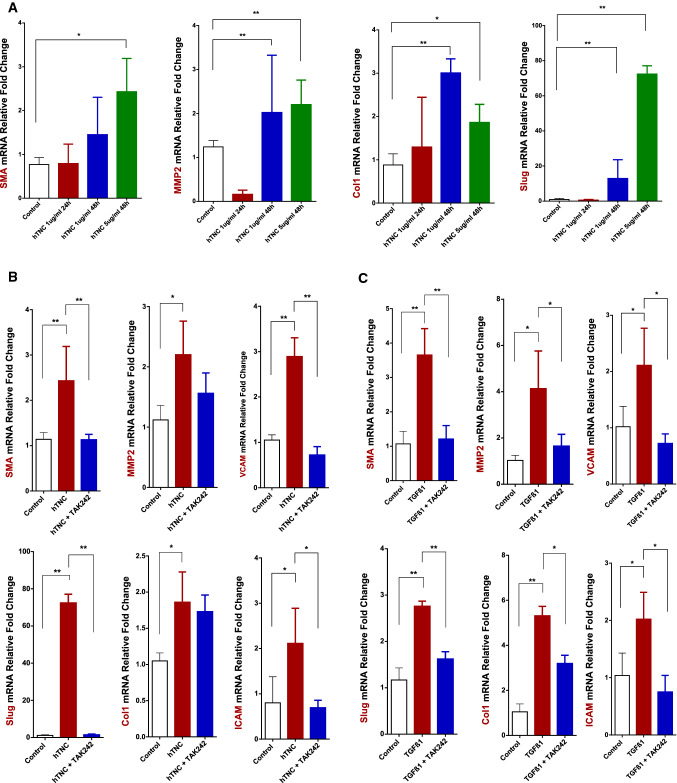
TNC and TLR4 influence on EMT induction in MVEC. αSMA, MMP2, Collagen1, and Slug mRNA expression levels measured by RT-qPCR in MVEC treated with different hTNC concentrations (1 and 5 µg/ml) and harvested after 24 or 48 h (*n* = 5) (a). αSMA, MMP2, VCAM-1, ICAM-1, Slug, and Collagen1 mRNA levels measured by RT-qPCR after pretreatment with TAK242 (50 mM) for 30 min and exposure to hTNC (5 µg/ml for 48 h) (*n* = 3) (B) or TGFβ1 (1 ng/ml for 96 h) (*n* = 3) (c). *p < 0.05 **p < 0.001

Since TNC expression at the valvular level was well correlated to the mitral valve increased thickness in ischemic MR animals, we wanted to investigate by which mechanism TNC was potentially contributing to leaflet remodeling. A previous study [7] already showed the crucial role of EMT in valvular remodeling following MI. Therefore, we wanted to test whether TNC could induce EMT in MVEC.

After exposing MVEC to different hTNC concentrations, a significant increase in α-SMA mRNA expression was only observed in cells treated with hTNC at 5 µg/ml for 48 h (*p* = 0.03). EMT-associated transcription factor Slug was already significantly increased at lower doses of hTNC (1 µg/ml) after 48 h. Collagen1 and MMP2 mRNA expression was upregulated after 48 h of exposure to either 1 or 5 µg/ml of hTNC. These results suggest that TNC might be able to induce EMT in MVEC.

### TLR4 inhibition effect on TNC and TGFβ1-mediated EMT in MVEC

After showing TNC potential to induce EMT in MVEC, we wanted to further explore TNC-mediated EMT mechanisms. Since TNC was shown to be an endogenous activator of TLR4 [20], we tested whether TNC would still have the same effect after pretreating the cells with a TLR4 inhibitor (TAK242). Slug and α-SMA were both significantly downregulated after TLR4 inhibition compared to the TNC treatment alone group (Fig. 6b). Besides, TNC induced an upregulation in vascular cell adhesion molecule-1 (VCAM-1) and intracellular adhesion molecule-1 (ICAM-1) expression, which was reduced by TLR4 inhibition.

TGFβ1 is known to induce EMT in MVEC [1] and was used in parallel to our experiments as positive EMT control. Interestingly, the involvement of TLR4 has not been shown in this process yet. After pretreating MVEC with TAK242 (50 nM for 30 min), the cells were exposed to TGFβ1 (1 ng/ml) and were harvested after 96 h. TGFβ1 induced upregulation in α-SMA expression as well as VCAM-1, ICAM-1, Collagen1, and MMP2, which was prevented by inhibiting TLR4. TGFβ1 induced an upregulation in Slug, an EMT transcription factor. This effect was reversed by TLR4 inhibition pretreatment (Fig. 6c).

TGFβ1 is not known to be a TLR4-activator contrary to TNC. We therefore hypothesized that TGFβ1 effect on MVEC involved TNC, which was the one acting on TLR4 to promote EMT. After exposing MVEC to TGFβ1 (1 ng or 10 ng/ml) for 3–6 h as well as 1 ng/ml for 96 h, we analyzed TNC gene expression. TNC mRNA expression was upregulated only at 3 h either with 1 or 10 ng/ml of TGFβ1 (Supplementary Fig. 1).

## Discussion

This study describes for the first time the possible contribution of TNC to valvular remodeling. Indeed, we show an upregulation of TNC in the posterior mitral leaflet in two large animal models of ischemic MR as well as patients’ samples with ischemic MR. We also show TNC potential in-vitro to activate MVEC through EMT into a myo-fibroblastic phenotype, which was mediated by TLR4. This cellular activation on the long term promotes fibrosis and valvular remodeling, which ultimately compromises leaflet flexibility [7]. These new insights into the pathophysiological mechanisms of valvular remodeling in ischemic MR may provide novel therapeutic targets to limit ischemic MR.

Our results show that after 6 weeks, the posterior leaflet under tethering forces generated by a localized infarct to the PMPM had an increased length, but was also thicker compared to healthy animals. Whereas in the sheep group, after subjecting the mitral valve apparatus to the same stress for a longer period (6 months), we noticed that there was only an increased leaflet thickness without an increase in the leaflet length. The increase in the leaflet length as observed in the pigs after 6 weeks could be explained by either a passive mechanical stretch or an attempt to adapt to MR. Indeed, previous studies showed that mitral leaflets adapt to LV enlargement by increasing their surface to insure an efficient coaptation and limit MR incidence [23]. However, this adaptive ability is attenuated after MI as shown recently in an ovine animal model [16]. Furthermore, increased leaflet thickness might be a response to preserve leaflet integrity and prevent its rupture, but if excessive, it might also lead to stiffer leaflets contributing to worsening valve restriction, which might explain our observations in the sheep model after 6 months with an increase in leaflet thickness but not in length. In fine, this might reflect the inadequacy of the adaptive mechanisms triggered initially to maintain a sufficient leaflet growth to limit MR.

We also show in these experiments that TNC plays a pivotal role in valvular remodeling following ischemic MR and stimulates EMT in isolated MVEC. This cellular process, where endothelial cells express α-SMA, was inhibited by TLR4 blockade in an in-vitro setting.

TNC is an extracellular matrix (ECM) glycoprotein. While it is normally absent or found in low amounts in the adult heart, TNC is highly expressed during embryonic development, inflammation, and wound healing [12]. Besides, it has been demonstrated that TNC expression was closely correlated to EMT in cardiac cushions [2]. Its expression is upregulated by mechanical stress [25] but also by different cytokines and growth factors such as TGFβ1 [13].

TNC upregulation in our ischemic MR models may have resulted from mechanical stress [25]. As an adaptation to tethering forces and flow turbulences caused by ischemic MR, TNC may at first intervene as a compensatory mechanism to protect the valve from tearing by augmenting ECM resilience as this has been observed at the myocardial level as a response to pressure overload [22]. However, its persistence becomes counterproductive, resulting in increased valvular thickness and stiffness instead of increasing the leaflet area and, therefore, jeopardizing MV coaptation. Indeed, previous studies showed the ability of the mitral valve to adapt different responses to mechanical stress in the presence or absence of MI unraveling the valvular remodeling with an increased valvular thickness as a possible mechanism to the appearance of secondary MR in the setting of MI [6, 7, 16]. Even though this study was based on animal models, but to prove the translational potential of our findings, we confirmed TNC expression in mitral valves from ischemic MR patients despite the more advanced disease stage, suggesting that TNC is part of the persisting remodeling process.

As TNC is known to be involved in inflammatory and fibrotic processes, we explored the presence of inflammatory process through TLR4 expression at the valvular level. Indeed, we observed in the animal models an TLR4 expression upregulation in leaflets from ischemic MR groups. TLR4 is a member of the Toll-like receptor family. It can be activated either by exogeneous stimuli or as a response to internal damage cues [9, 18]. Its activation leads to a cascade of inflammatory responses, which represents the first line of innate host defense. TLR4 expression at the valvular level was significantly higher after 6 weeks. Even though TLR4 expression persisted at 6 months, the percentage of cells expressing TLR4 was lower compared to the early time-point. This would suggest that the inflammatory process activated at an early time-point resulted in a counterproductive fibrosis as shown by the leaflet thickness and length evolution in these animals. TLR4 persistence at lower amounts might have contributed to the fibrotic process, resulting in thicker leaflets with no increase in their area. TLR4 can be expressed by various cell types including macrophages. Future studies will be needed to clarify whether macrophages have a role in valvular remodeling.

Besides, TNC expression in the mitral leaflets in the animal models, we observed α-SMA staining in the endothelium (Fig. 4a, black arrows). Endothelial cells in their basal state express CD31 but no α-SMA. We also noticed CD31 expression in the valvular interstitium (Fig. 4b, black stars) suggesting a migration of endothelial cells. Taken together, these results point to EMT of MVEC. This is consistent with previous work, where EMT load was shown to be closely correlated to the valvular remodeling secondary to tethering and was exacerbated in the presence of MI [4, 7].

We sought then to explore whether TNC could contribute to the EMT phenomenon observed at the valvular level. Our cell experiments demonstrated the capacity of TNC to induce EMT through Slug, which was limited after TLR4 inhibition. Indeed, TNC is known to act as damage-associated molecular patterns (DAMP) to activate innate immunity mediated by TLR4 [17, 20].

Interestingly, TGFβ1-mediated EMT was also downregulated after TLR4 inhibition in-vitro. Even though TGFβ1 is not known to be a TLR4 agonist, our results could be explained by the observed TNC upregulation after TGFβ1 stimulation, which may have further induced EMT in MVEC and was blocked after TLR4 inhibition. These results suggest that TGFβ1-mediated EMT involves TNC stimulation of TLR4. TNC expression was upregulated early after exposing the cells to TGFβ1 but no longer at later timepoints.

Further work will be needed to better understand TLR4 involvement in TGFβ1-mediated EMT in MVEC. Previous work has shown an intersection between TGFβ1 and TLR4 networks [9]: TLR4 stimulation by ECM components such as TNC secreted after TGFβ1 stimulation may have downregulated activin membrane-bound inhibitor (BAMBI), enhancing TGFβ1 signaling [10]. This TGFβ1 and TLR4 network intersection could explain our results, where TNC was upregulated at an early time-point to stimulate TLR4 downregulating BAMBI, thus facilitating TGFβ1 signaling.

### Clinical and therapeutic perspectives

Mitral valve ability to adapt to LV remodeling to limit MR has been shown. Nevertheless, ischemic milieu have a negative impact on the adaptation ability of the valve with an exuberant EMT induced by TGFβ1 [6, 7]. Our new observations suggest that TNC plays a role in altering valvular flexibility by enhancing EMT, which can be reduced by TLR4 blockade. Better understanding the mechanisms involved in leaflet fibrosis and excess EMT is crucial to modulate the cellular processes and guide them towards increasing leaflet area without compromising their flexibility and their coaptation. Future studies may consider the local delivery of a TLR4 inhibitor to limit EMT and subsequent valvular remodeling. Furthermore, TNC antagonism could also be of interest in this setting. Even though more experiments will be required to elucidate by which mechanisms TLR4 affects EMT, our study sets novel therapeutic targets to ischemic MR.

### Limitations

For this study, we used two different species for the short- and long-term ischemic MR model. Even though we started establishing our initial model in pigs, we could no longer use the same animal species for a 6-month follow-up. Indeed, swine models are limited by the rapid growth rate.

The in-vitro studies conducted on MVEC used primary cells from both atrial and ventricular leaflet endothelium without distinction. MVEC from different leaflet sides can have a different biological response. Furthermore, in-vitro behavior of MVEC can be different from what would be observed in their natural environment. Therefore, the results obtained after TLR4 blockade will have to be tested in an animal model.

Native endothelial cells population comprises MVEC at different stages of activation in different proportions to maintain the valvular hemostasis. Clonal isolation of MVEC allows to select specific endothelial cell sub-population and a more-controlled experimental setting. We did not used monoclonal cultures in our in-vitro study as this would have resulted in cellular senescence and dedifferentiation before reaching a suitable cell number.

TNC was demonstrated to be upregulated at valvular level in both animal models as well as in patients’ samples. However, further studies are warranted to clarify its source.

Even though our animal models were designed to mimic as closely as possible ischemic MR encountered in patients, these models are limited by the severity of the MR generated (moderate) as well as the evolution time.

## Electronic supplementary material

Below is the link to the electronic supplementary material.

Supplementary file1 (DOCX 24 kb)

Supplementary file1 (PDF 33 kb)
