# Supplementary material for: Tenascin C promotes valvular remodeling in two large animal models of ischemic mitral regurgitation
Source: Basic Res Cardiol. 2020 Dec 1;115(6):76. doi: 10.1007/s00395-020-00837-5 (PMC7716900; doi:10.1007/s00395-020-00837-5)
Supplement: Supplementary file 1 — Supplementary file1 (DOCX 24 kb) [file 395_2020_837_MOESM1_ESM.docx]

**Supplementary Figures Legends:**

**Supplementary Figure 1**

TNC mRNA levels measured by RT-qPCR in MVEC treated with different TGFβ1 concentrations (1 or 10ng/ml) and harvested after 3, 6 or 96 hours (n=3)

**Supplementary Table 1**. Anesthesia regiments used for pigs and sheep for both the myocardial infarction procedure and the final examination.

|  | **Pigs** | | **Sheep** | |
| --- | --- | --- | --- | --- |
| **Premedication** | Ketamine | 25mg/kg IM | Atropin | 1 mg IM |
|  | Midazolam | 0,75mg/kg | Ketamin | 2 – 4 mg IM |
|  |  |  | Detomidin | 0.01 – 0.02mg/kg IM |
| **Induction** | Propofol | 2.5 – 5mg/kg IV | Propofol | 2.5 – 5 mg/kg IV |
|  | Fentanyl | 0.1mg IV | Fentanyl | 0.1mg IV |
| **Maintenance** | Fentanyl | 0.01mg/kg/h | Isofluran | 1 – 2% |
|  | Propofol | 10 – 20mg/kg/h | Fentanyl | 0.01mg/kg/h IV |
|  |  |  | Atracurium Besylate | 0.3mg/kg/h IV |

**Supplementary Table 2.** Primary antibodies used for immunohistochemistry

| **Name** | **Company** | **Order-No** | **Clonality** | **Host** | **Dilution** |
| --- | --- | --- | --- | --- | --- |
| CD31 | abcam | ab28364 | poly | rabbit | 1:50 |
| aSMA | Novusbio | NBP2-33006 | mono | mouse | 1:500 |
| TLR4 | abcam | ab22048 | mono | mouse | 1:100 |
| TNC | Merck | AB19011 | poly | rabbit | 20µg/ml |

| **Gene** | **Forward 5'-3'** | **Primer length (bp)** | **Reverse 5'-3'** | **Primer length (bp)** |
| --- | --- | --- | --- | --- |
| TNC | CACACACGAAATCACTGCTAAC | 22 | TTTTCCTTCTCTTGGGTGGCT | 21 |
| SMA | ACGGGTAGCCAGCATGAAAG | 20 | GCGGTTCAGATTTGGTGTGG | 20 |
| MMP2 | GAACCGTTGAAGGGAGGGGA | 20 | AGTCACTGCACAATGCCTCTC | 21 |
| COLL1 | TTAGGCAAGGAACAGAGCGG | 20 | GGGTTCGGAGGAAAGTCAGG | 20 |
| ICAM-1 | AACTTCTGCACCAGGTCTAACA | 22 | TTGAATGGATGAAGGAGCCC | 20 |
| VCAM-1 | AACTTCTGCACCAGGTCTACCA | 22 | TTGAATGGATGAAGGAGCCC | 20 |
| SLUG | GTGGATAGGCTTTCCCTCG | 19 | CTTCCCACCCTGCTACTCG | 19 |
| TBP | AACAGTTCAGTAGTTATGAGCCAGA | 25 | AGATGTTCTCAAACGCTTCG | 20 |
| HMBS | TTCATTCCCTCAAGGACCTG | 20 | GGGGTGAAAGACAACAGCAT | 20 |

**Supplementary Table 3:** Primers used for the RT-qPCR

**Supplementary Table 4.** Echocardiographic parameters at baseline and final examination (at 6 weeks for the pigs and 6 months for the sheep)

|  | **Pigs** | | **Sheep** | |
| --- | --- | --- | --- | --- |
|  | **Baseline** | **6 weeks** | **Baseline** | **6 Months** |
| **EF%** | 63 ± 3 | 47 ± 3* | 60 ± 3 | 42 ± 2* |
| **EDD mm** | 50.31 ± 4.58 | 62.12 ± 3.93* | 47.67 ± 3.44 | 63.47 ± 3.38* |
| **ESD mm** | 29.86 ± 2.96 | 42.53 ± 5.78* | 28.44 ± 2.75 | 41.75 ± 6.01* |
| **EDV ml** | 84.71 ± 8.53 | 209.71 ± 7.68* | 74 ± 4.32 | 212 ± 15.41* |
| **ESV ml** | 38.14 ± 8.65 | 95.42 ± 21.61* | 34.5 ± 5.03 | 82.33 ±7.97* |
| **LAS cm^2^** | 7.46 ± 0.48 | 14.87 ± 1.5* | 7.87 ± 0.65 | 15.9 ±1.58* |
| **LAV ml** | 14.57 ± 1.05 | 58 ± 16.86* | 13.83 ±1.95 | 56.83 ±4.1* |
| **IJA %** | 3.85 ± 1.21 | 23.9 ± 2.74* | 3.23 ± 1.84 | 22.89 ±2.58* |
| **Vena contracta mm** | 0.53 ± 0.15 | 3.49 ± 0.27* | 0.47 ± 0.25 | 3.98 ± 0.24* |
| **Tenting area cm²** | 0.71 ± 0.09 | 2.63 ± 0.7* | 0.72 ± 0.07 | 3.02 ± 0.81* |
| **Tenting height mm** | 3.27 ± 0.45 | 12.03 ± 3.18* | 3.28 ± 0.31 | 13.81 ± 3.72* |

The values are expressed as mean ± SD. EF: ejection fraction, EDD: end-diastolic diameter, ESD; end-systolic diameter, EDV: end-diastolic volume, ESV: End-systolic volume, LAS: left atrium surface, LAV: left atrium volume, IJA: indexed jet area to left atrium surface. *p<0.01
