# Supplementary figures and images for: Tenascin C promotes valvular remodeling in two large animal models of ischemic mitral regurgitation
Source: Basic Res Cardiol. 2020 Dec 1;115(6):76. doi: 10.1007/s00395-020-00837-5 (PMC7716900; doi:10.1007/s00395-020-00837-5)

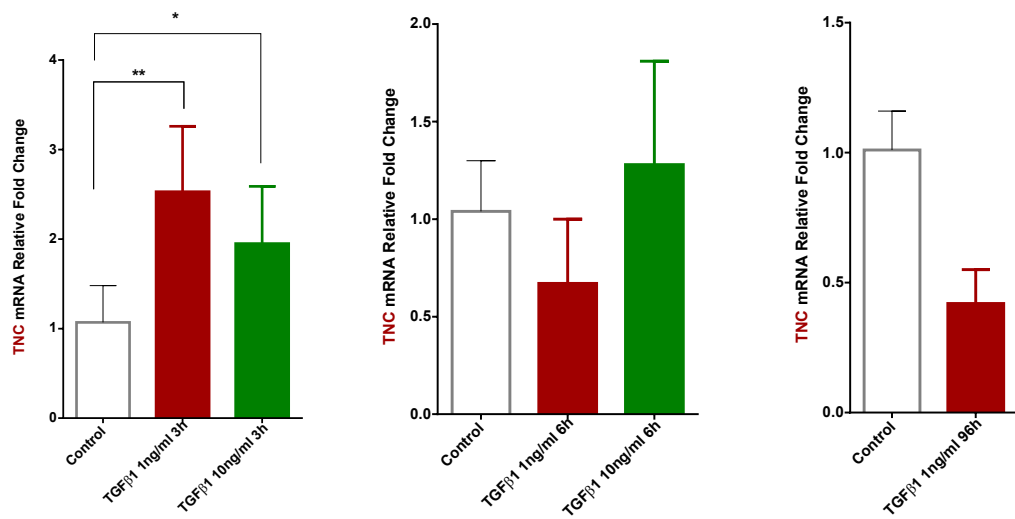

Supplement: Supplementary file 2 — Supplementary file1 (PDF 33 kb) [file 395_2020_837_MOESM2_ESM.pdf]
